# Optimisation and Application of a Novel Method to Identify Bacteriophages in Maternal Milk and Infant Stool Identifies Host-Phage Communities Within Preterm Infant Gut

**DOI:** 10.3389/fped.2022.856520

**Published:** 2022-04-26

**Authors:** Gregory R. Young, Wen C. Yew, Andrew Nelson, Simon H. Bridge, Janet E. Berrington, Nicholas D. Embleton, Darren L. Smith

**Affiliations:** ^1^Faculty of Health and Life Sciences, Northumbria University, Newcastle upon Tyne, United Kingdom; ^2^Hub for Biotechnology in the Built Environment, Northumbria University, Newcastle upon Tyne, United Kingdom; ^3^Translational and Clinical Research Institute, Faculty of Medical Sciences, Newcastle University, Newcastle upon Tyne, United Kingdom; ^4^Newcastle upon Tyne Hospitals National Health Service Foundation Trust, Newcastle upon Tyne, United Kingdom; ^5^Population Health Sciences Institute, Faculty of Medical Sciences, Newcastle University, Newcastle upon Tyne, United Kingdom

**Keywords:** preterm/full term infants, microbiota, bacteriophage (phage), laboratory method, breast milk, microbiome, dysbiosis

## Abstract

Human milk oligosaccharides, proteins, such as lactoferrin, and bacteria represent just some of the bioactive components of mother's breast milk (BM). Bacteriophages (viruses that infect bacteria) are an often-overlooked component of BM that can cause major changes in microbial composition and metabolism. BM bacteriophage composition has been explored in term and healthy infants, suggesting vertical transmission of bacteriophages occurs between mothers and their infants. Several important differences between term and very preterm infants (<30 weeks gestational age) may limit this phenomenon in the latter. To better understand the link between BM bacteriophages and gut microbiomes of very preterm infants in health and disease, standardised protocols are required for isolation and characterisation from BM. In this study, we use isolated nucleic acid content, bacteriophage richness and Shannon diversity to validate several parameters applicable during bacteriophage isolation from precious BM samples. Parameters validated include sample volume required; centrifugal sedimentation of microbes; hydrolysis of milk samples with digestive enzymes; induction of temperate bacteriophages and concentration/purification of isolated bacteriophage particles in donor milk (DM). Our optimised method enables characterisation of bacteriophages from as little as 0.1 mL BM. We identify viral families that were exclusively identified with the inclusion of induction of temperate bacteriophages (*Inoviridae*) and hydrolysis of milk lipid processes (*Iridoviridae* and *Baculoviridae*). Once applied to a small clinical cohort we demonstrate the vertical transmission of bacteriophages from mothers BM to the gut of very preterm infants at the species level. This optimised method will enable future research characterising the bacteriophage composition of BM in very preterm infants to determine their clinical relevance in the development of a healthy preterm infant gut microbiome.

## Introduction

It is widely accepted that mother's breast milk (BM) is the gold standard of nutritional care for newborn infants (1, 2) due to the myriad of beneficial effects of BM for newborn infant growth and development (3, 4). Various studies have characterised the components present in BM, including human milk oligosaccharides (5), bioactive proteins, such as lactoferrin (6, 7), and bacteria (8–10), to elucidate potential modes of the improved health outcomes in infants who receive BM. There are several key differences between term and preterm infants, including length of hospitalisation and exposure of preterms, to necessary clinical interventions that impact microbiome development (11). These include chemical interventions, such as antibiotics and antivirals, or mechanical interventions, such as invasive catheters and feeding tubes, as well as exposure to microbes resident within the neonatal unit environment. In addition to these neonate-specific factors, evidence has emerged of longitudinal change to the nutritional composition of BM particularly the quantity and quality of proteins and lipids. Preterm BM, therefore, differs substantially from that of term BM, both in nutritional and microbial composition.

Another bioactive component of BM is the bacteriophage content. Initial analysis of vertical bacteriophage transmission between BM and the neonatal GI tract performed by Breitbart et al. (12) in 2008 found no shared bacteriophages between the single mother-infant pair studied despite large quantities of identifiable virus in infant stool. Since this pioneering study improvements in metagenomic sequencing technology and bacteriophage genome databases have facilitated further investigations. In 2017, Duranti et al. (13) reported the presence of *Bifidobacterium* phages in both mother's BM and their infant's stools demonstrating the potential of bacteriophage transmission in term mother-infant pairs. Pannaraj et al. (14) compared alpha and beta diversity of BM and stool samples from a separate cohort of term infants, finding a greater alpha diversity in BM viromes than stools as well as several shared taxonomic features between BM and stool from the same mother-infant pairs. However, these taxonomic analyses were capped at the family level which limits our ability to determine whether there is a transmission of bacteriophages from mother to infant *via* BM. A comparison between the virome of BM given to infants delivered at term or preterm found contrasting taxonomic compositions (15). This study identified several bacteriophage species with host specificity for common skin commensals. Preterm infants enrolled in the study were a minimum of 32 weeks gestation at birth although BM bacteriophages were not compared with infant stool bacteriophages. A knowledge gap still exists to elucidate bacteriophage transmission between BM and neonatal stool in very preterm (<30 weeks gestation) neonates.

Although bacteriophages have now been revealed as one of the active components of BM, understanding their full impact in preterm BM, including their interactions with microbes in the preterm infant GIT, remains enigmatic. A major obstacle to overcome when interpreting the results of each study investigating bacteriophages in BM is the methodological differences between studies (12, 14, 16). Indeed, some methods appear to have limitations that lead to selection bias during viral isolation (17). For instance, the application of glass beads during sample homogenisation can reduce non-enveloped bacteriophages, membrane filtration with 0.2 and/or 0.45 μM filters may fail to recover giant viruses, and caesium chloride (CsCl) density-gradient ultracentrifugation fails to recover enveloped bacteriophages or those within particular buoyant densities (17). Such contrasting approaches in the literature prove an even greater issue when investigating preterm infant microbiome because sample volumes are so small and are therefore extremely precious.

This study aims to develop a robust method for isolation of bacteriophages from the smallest possible volumes of BM from mothers to very preterm infants to promote accurate characterisation of these communities in future studies. We utilize pasteurised donor milk (DM) to validate bacteriophage isolation procedures, then apply the validated method to BM and neonatal stools collected from 4 mother-infant pairs to demonstrate its utility in identifying vertical transmission of bacteriophage species between clinical BM and stool samples.

## Materials and Methods

### Development and Validation of Bacteriophage Isolation Protocol

All laboratory work was conducted in a Class II safety cabinet using nuclease-free or sterilised consumables and equipment. PROLACT HM^®^ human donor milk (Prolacta Bioscience Inc., USA) was used for method validation experiments. Donor milk (DM) was stored at −80°C until the day of processing, when samples were defrosted at 4°C, gently mixed by inversion and dispensed into 1 mL aliquots. These were then further aliquoted, on ice, into 0.1, 0.2, 0.4, and 0.6 mL volumes to validate the required sample volume for bacteriophage isolation and DNA extraction. Six replicates of each sample volume were processed. DM aliquots were homogenised with cold sterile 1x PBS in a ratio of 1:5 (Supplementary Table 1).

### Experiment 1: Sedimentation of Microbial Cells With Increased Centrifugation Speeds

To assess the effect of centrifugal force in separating microbial cells for the induction of temperate bacteriophages, eight replicates of PBS-homogenised DM were split into three centrifugation groups (Supplementary Figure 1). In each group, samples were centrifuged at 400 xG at room temperature for 10 minutes to separate the DM lipid and skimmed milk, as previously described (18). The DM lipids were discarded, while the skimmed milk was centrifuged at 10, 15, and 20 k xG at 4°C for 10 min. The resulting microbial cell pellets were incubated with norfloxacin (1 μg/mL) at 37°C for 1 h to induce temperate bacteriophages, followed by centrifugation at 15 k xG at 4°C for 10 min to remove remaining microbial cell debris (19). Supernatants resulting from the first centrifugation (i.e., free bacteriophages) and the second centrifugation (i.e., induced temperate bacteriophages) were subjected to DNase (2 U/mL) and RNase (0.5 U/mL) treatments to remove human and microbial chromosomal DNA, as previously described (19). DNA of the isolated bacteriophages were then extracted using QIAamp MinElute Virus Spin Kit (QIAGEN, Germany), following the manufacturer's protocol. Equal volumes of autoclaved 1x PBS were included as a kit negative control in every extraction batch.

### Experiment 2: Release of Bacteriophages in DM and Milk-Lipids With Digestive Enzymes

Digestive enzymes similar to those in infant guts (20) were used to hydrolyse milk fat globule membrane (MFGM) and release bacteria and bacteriophages that are associated with the MFGM. For hydrolysis of DM (Supplementary Figure 2), four replicates of PBS-homogenised DM were incubated with digestive enzymes (0.4 mg/mL of pepsin and 0.86 mg/mL of lipase) at 37°C and shaking at 350 rpm for an hour. Hydrolysed samples were centrifuged at 400 xG at room temperature for 10 min to separate the DM lipid and skimmed milk, as previously described (18). The resulting DM lipids were carefully removed and were discarded, whilst the skimmed milk was centrifuged at 10 k xG at 4°C for 10 min. As described in *Experiment 1*, the separated microbial cell pellets were then subjected to norfloxacin-mediated bacteriophage induction and removal of remaining microbial cell debris. Both supernatants of free and temperate bacteriophages were ready for the subsequent steps of DNase and RNase treatments and DNA extraction.

For hydrolysis of DM lipids (Supplementary Figure 3), four replicates of PBS-homogenised DM samples were centrifuged at 400 xG and room temperature for 10 min. The resulting DM lipids were carefully separated and homogenised in 1 mL of cold sterile 1x PBS. Four further DM samples were frozen at −20°C for 30 min following centrifugation before separating the DM lipids and homogenising in cold sterile 1x PBS. These frozen lipid samples were used to validate freezing the DM lipid fraction during bacteriophage isolation. PBS-homogenised lipid suspensions were incubated with digestive enzymes at 37°C and shaking at 350 rpm for an hour, then centrifuged at 10 k xG at 4°C for 10 min for the separation of free bacteriophages and microbial cell pellets. As described upfront in *Experiment 1*, the separated microbial cell pellets were then subjected to norfloxacin-mediated bacteriophage induction and removal of remaining microbial cell debris. Both supernatants of free and temperate bacteriophages were ready for the subsequent steps of DNase and RNase treatments and DNA extraction.

In addition, four replicates of PBS-homogenised DM samples without the input of digestive enzymes were processed as a control (Supplementary Figure 4).

### Experiment 3: Concentrating and Purifying Isolated Bacteriophages

Isolated bacteriophages from the DM (Supplementary Figures 2, 4), were subjected to further concentration steps using 10 % PEG8000 and purification steps using Centriprep^®^ 50 kDa filter devices (Merck Millipore, USA), as previously described (21, 22).

In addition, four replicates of PBS-homogenised DM samples without the application of 10 % PEG8000 and Centriprep^®^ 50 kDa filter devices were processed as a control (Supplementary Figure 4).

### Application of Validated Bacteriophage Isolation Protocol to Mother-Infant Pair Samples

The pilot study utilised BM and stool samples from a total of four very preterm (<30 weeks gestation) mother-infant pairs enrolled on the SERVIS study at the Neonatal Intensive Care Unit, Royal Victoria Infirmary, Newcastle upon Tyne, UK, as previously described (8, 23). Samples were collected with ethical permission granted by NRES Committee North East–Newcastle & North Tyneside 2 (10/H0908/39) and analysed with written parental consent. Expressed BM and stool samples were stored at −80°C until processing. Bacteriophage isolation and DNA extraction were performed using the optimised protocol in **Figure 4**.

### Bacteriophage Metagenome Sequencing

The concentration and purity of extracted bacteriophage DNA were determined using Qubit 2.0 Fluorometer (Thermo Fisher Scientific, USA) and NanoDrop™ 1000 Spectrophotometer (Thermo Fisher Scientific, USA), respectively. Nucleic acid sequencing was performed by NU-OMICS DNA Sequencing Research Facility (Northumbria University, UK) using the Nextera XT kit and V3 600 cycle chemistry on the Illumina MiSeq (validation study) and V2.5 300 cycle mid-output chemistry on the Illumina NextSeq (pilot study) (Illumina Inc., USA). Raw sequencing data are available at ENA (study accession PRJEB49989).

### Informatic Processing of Method Validation Data

Raw read data were de-multiplexed using MiSeq Reporter Software (Illumina, USA). Illumina adapter sequences were removed, sequences were trimmed with a sliding window (Q30) and any reads <50 nucleotides or >300 nucleotides were culled using *FastQC* (24) and *Trimmomatic* (25) in *Galaxy* (26). Trimmed paired and unpaired reads files were merged for further filtering with *BBTools* (27). Sequences identified in negative controls were culled from experimental samples. Low-complexity reads were removed using *BBmask* (entropy 0.7), and duplicated reads were removed with *BBduk*. Resulting reads were mapped to the human genome (GRCh38) using *BBmap* with high precision but low sensitivity parameter. Unmapped reads were aligned against NCBI viral reference database (accessed 16/07/2021 at https://ftp.ncbi.nlm.nih.gov/refseq/release/viral/) using the *blastn* option in BLAST (evalue 10-5, best-hit overhang 0.1, best-hit score edge 0.1). BLAST output reads were uploaded to *MEGAN* Community Edition (28), and taxonomy assignment was performed using a modified lowest common ancestor (LCA) algorithm (min score 100, E-value <10-5, min percent identity 80, min support 1, weighted 80). Enrichment of viral sequences in metagenomes was quantified using *viromeQC* (29).

### Informatic Processing of Clinical Pilot Study

Raw paired end.*fastq* files were quality filtered with *fastp* (30) employing a 5 nucleotide sliding window and a minimum qscore >25. Reads <100 nucleotides were discarded. *Kneaddata* within the *biobakery* environment (31) was used to remove human reads. Contigs were assembled from cleaned reads using the default kmer steps in *Megahit* (32). Contigs with <5x coverage were culled with *bwa mem* (33)*, samtools* (34), *bamtools* (35) and *bedtools* (35). *Virsorter2* (36) was used to identify dsDNA phage, nucelocytoplasmic large-DNA viral, RNA viral and ssDNA viral contigs from the >5x contigs and *CheckV* (37) was used to estimate the completeness of viral assembled genomes. Taxonomic classification of >5x coverage viral contigs was performed with *Kraken2* (38) by alignment to pre-built viral databases (accessed 04/01/2022, at https://benlangmead.github.io/aws-indexes/k2). Per-sample abundance of each classified viral contig was calculated using *Bracken* (39).

#### Statistical Analyses

Validation of bacteriophage isolation experiments was based on the concentration of DNA in nucleic acid extracts as well as the recovered viral richness and Shannon diversity index. R studio was used for all statistical analysis (40). The taxonomic richness and Shannon diversity measures of alpha diversity were calculated with the *vegan* package (41). Richness was calculated at a rarefied feature count below the minimum sample level where possible. Comparisons of continuous variables were performed by the Kruskal-Wallis test. Where significant (*P* > 0.05) differences were identified across experiment parameters the pairwise Wilcoxon test was used to perform pairwise comparisons between classes. Count and categorical data were compared by the Mann-Whitney test. In cases of multiple hypothesis testing *P*-values were adjusted using the Bonferroni method. The *phyloseq* (42) and *ggplot* (43) packages were used for data manipulation and visualisation.

## Results

### Validation of Bacteriophage Isolation

#### Viral Composition of DM Samples

A median of 2.99 x 10^5^ (IQR 8.82 x 10^4^ – 4.81 x 10^5^) raw reads were generated from 78 pasteurised DM samples (Supplementary Table 1). A total of 119 viral genera belonging to 18 families were identified (Supplementary Table 2). Bacteriophages accounted for 75 % of the total taxonomic reads. Predominant bacteriophage families included *Siphoviridae* (48%), *Myoviridae* (19%), *Podoviridae* (6%) and *Herelleviridae* (1%). The assigned bacteriophages are mainly associated with bacterial hosts *Escherichia* (17%), *Salmonella* (12%), *Enterococcus* (12 %), *Klebsiella* (7%), *Staphylococcus* (3%), *Pseudomonas* (3%), *Enterobacter* (3%), *Acinetobacter* (1%), *Proteus* (1%), *Erwinia* (1%), *Lactococcus* (1%) *Shigella* (1%) and *Stenotrophomonas* (1%) based on sequence homology. Unclassified bacteriophages constituted a further 1% of total reads.

#### Effect of Sample Volume on DM Bacteriophage Communities

**Figure 1:**
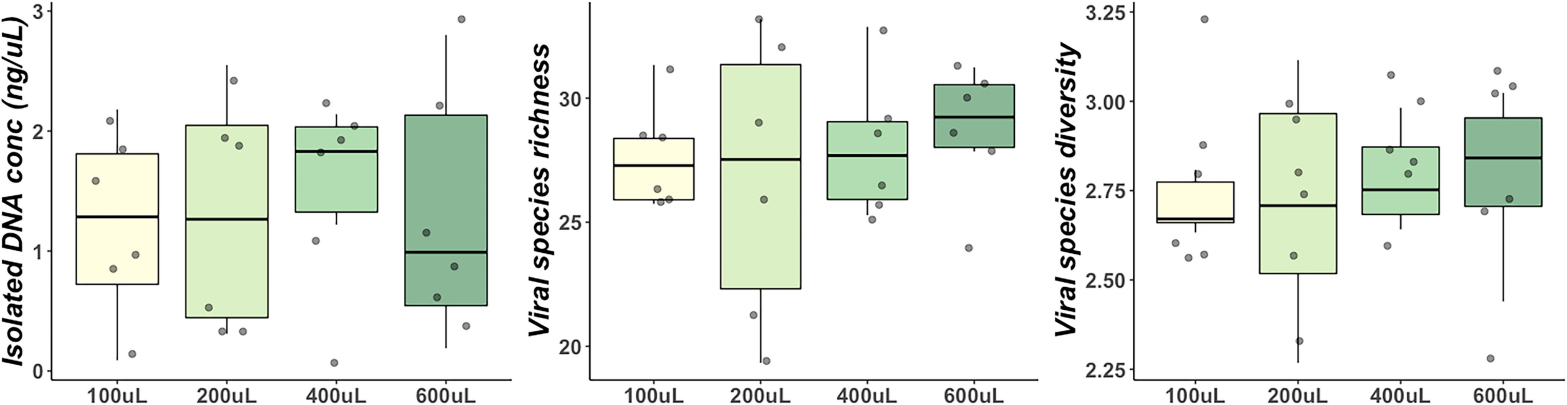
Comparisons of DNA concentration, genus richness and Shannon diversity between different volumes of milk samples. Boxes represent the 1st and 3rd quartiles. Middle lines represent the median. Whiskers extend to the full range of data. Points represent individual samples.

No significant difference in extracted DNA concentration (ng/μL) (*P* = 0.96), genus richness (*P* = 0.91) or Shannon diversity (*P* = 0.86) was observed between DM sample volumes 0.1, 0.2, 0.4, and 0.6 mL (Figure 1).

#### Centrifugal Separation, Inclusion of Digestive Enzymes and Concentration/Purification of Viral Particles

**Figure 2:**
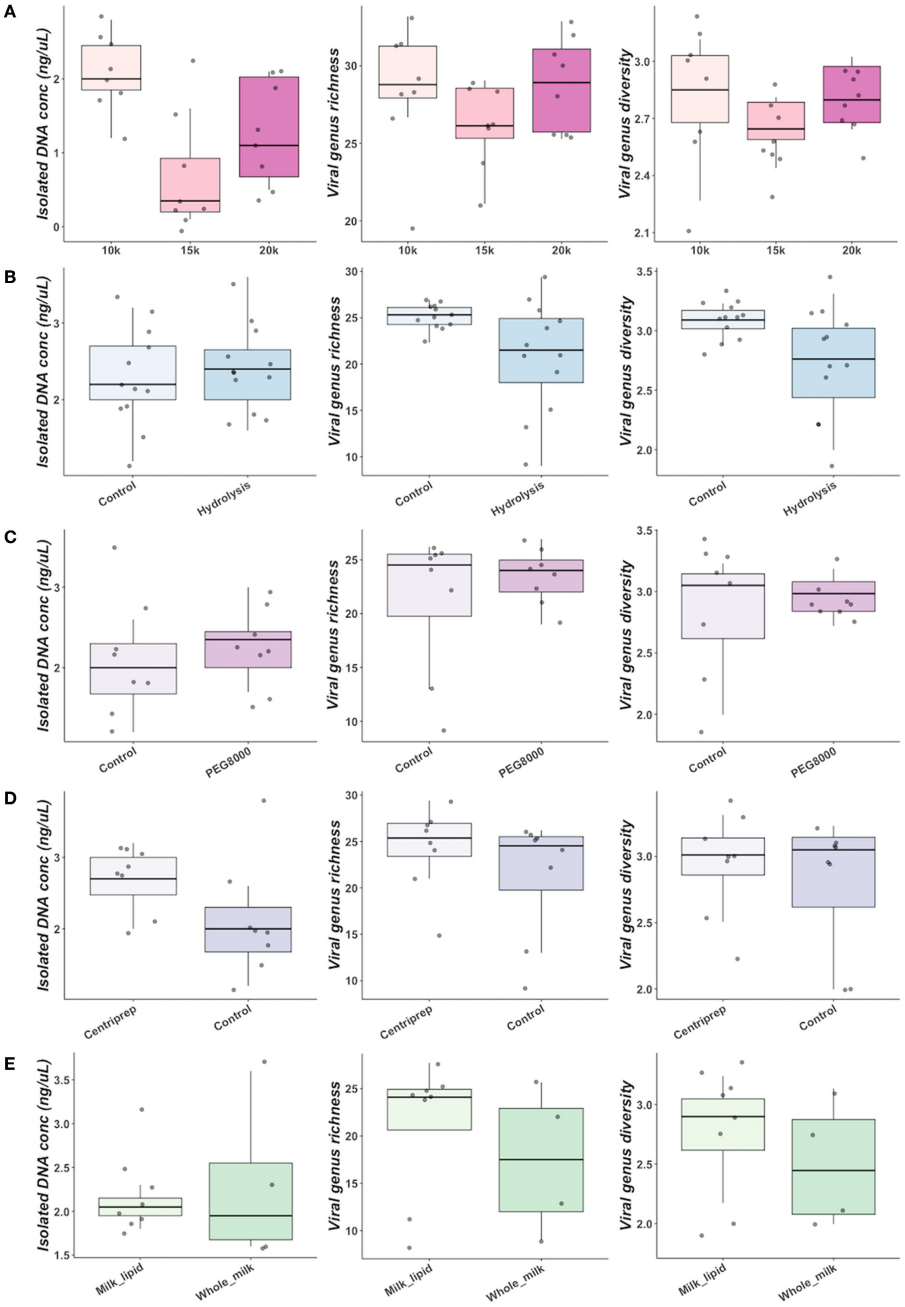
Comparisons of DNA concentration, genus richness and Shannon diversity between centrifugal spin speeds for size-based separation **(A)**, addition of digestive enzymes for milk hydrolysis **(B)**, concentration of bacteriophage particles with PEG8000 **(C)**, filtration of bacteriophage with Centriprep devices **(D)**, and utilization of different milk fractions **(E)**.

No significant differences in genus richness (*P* = 0.26) or Shannon diversity (*P* = 0.09) was observed in DM with increased centrifugation speed for microbial cell pelleting (Figure 2A). DNA concentration was significantly affected by centrifugation speed (*P* = 0.01) with significantly greater DNA concentrations observed in samples spun at 10 k xG (median 2.0 ng/μL) than at 15 k xG (median 0.35 ng/μL) (pairwise Wilcoxon: *P* = 0.02) (Figure 2A). Significantly lower Shannon diversity (*P* = 0.03) was observed in DM hydrolysed with digestive enzymes for lipid hydrolysis despite no difference in genus richness (*P* = 0.09) or DNA concentration (*P* = 0.54) (Figure 2B). The application of PEG8000 for concentration of isolated bacteriophages had no significant impact on DNA concentration (*P* = 0.32), genus richness (*P* = 0.92) or Shannon diversity (*P* = 0.92) of DM (Figure 2C). Concentrating and purifying bacteriophage isolates with Centriprep^®^ 50 kDa filter devices had no significant impact on observed genus richness (*P* = 0.25), or Shannon diversity (P = 1.00) (Figure 2D), but did marginally increase DNA concentration (*P* = 0.06) (Figure 2D). Separation of milk lipids from whole DM yielded no significant change in observed genus richness (*P* = 0.50), shannon diversity (*P* = 0.50), or isolated DNA concentration (*P* = 0.93) (Figure 2E).

**Figure 3:**
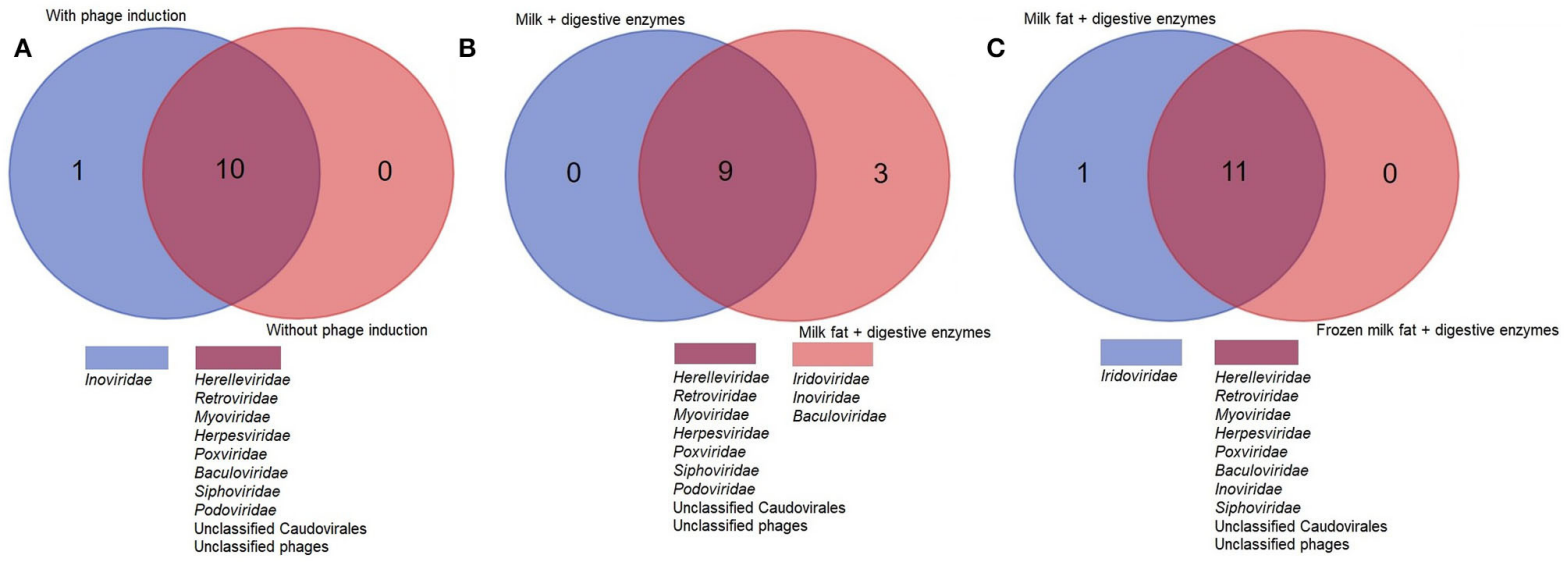
A higher viral richness was accounted for in samples at which **(A)** Norgen kit was used for DNA extraction, **(B)** norfloxacin-mediated phage induction was included or **(C)** digestive enzymes were added to the milk fat. The numbers within Venn diagrams represent the number of assigned families.

Further optimisation of commercially available nucleic acid extraction kits found comparable results for both QIAamp MinElute Virus Spin Kit (QIAGEN, Germany) and Norgen Phage DNA Isolation Kits (Norgen Biotek Corp, Canada). Both yielded comparable bacteriophage richness and diversity (Supplementary Table 4, Supplementary Figure 5) (Figure 3A).

Norfloxacin-mediated induction of bacteriophages enabled identification of *Inoviridae*, which were not seen in any non-induced samples. Although hydrolysis with digestive enzymes had no significant impact on alpha diversity indices or isolated DNA concentration, an improved recovery of lipid-associated viruses, enabling exclusive identification of *Iridoviridae* and *Baculoviridae* was observed in samples subjected to lipid hydrolysis (Figure 3B). Freezing the lipid layer before separation enabled identification of *Baculoviridae* and *Inoviridae* but not *Iridoviridae*, suggesting lipid hydrolysis may be essential to isolate this family.

Unlike the pasteurised DM used here for method validation, bio-banked BM samples do not undergo pasteurisation, which disrupts interactions between DM fat globule membranes. We found that when processing bio-banked BM samples an extra step of intense horizontal agitation for 10 min at room temperature as proposed by Brewster and Paul (44) caused lipid aggregation against the side of sampling tubes which made removal of BM lipids easier prior to digestive enzyme treatment.

**Figure 4:**
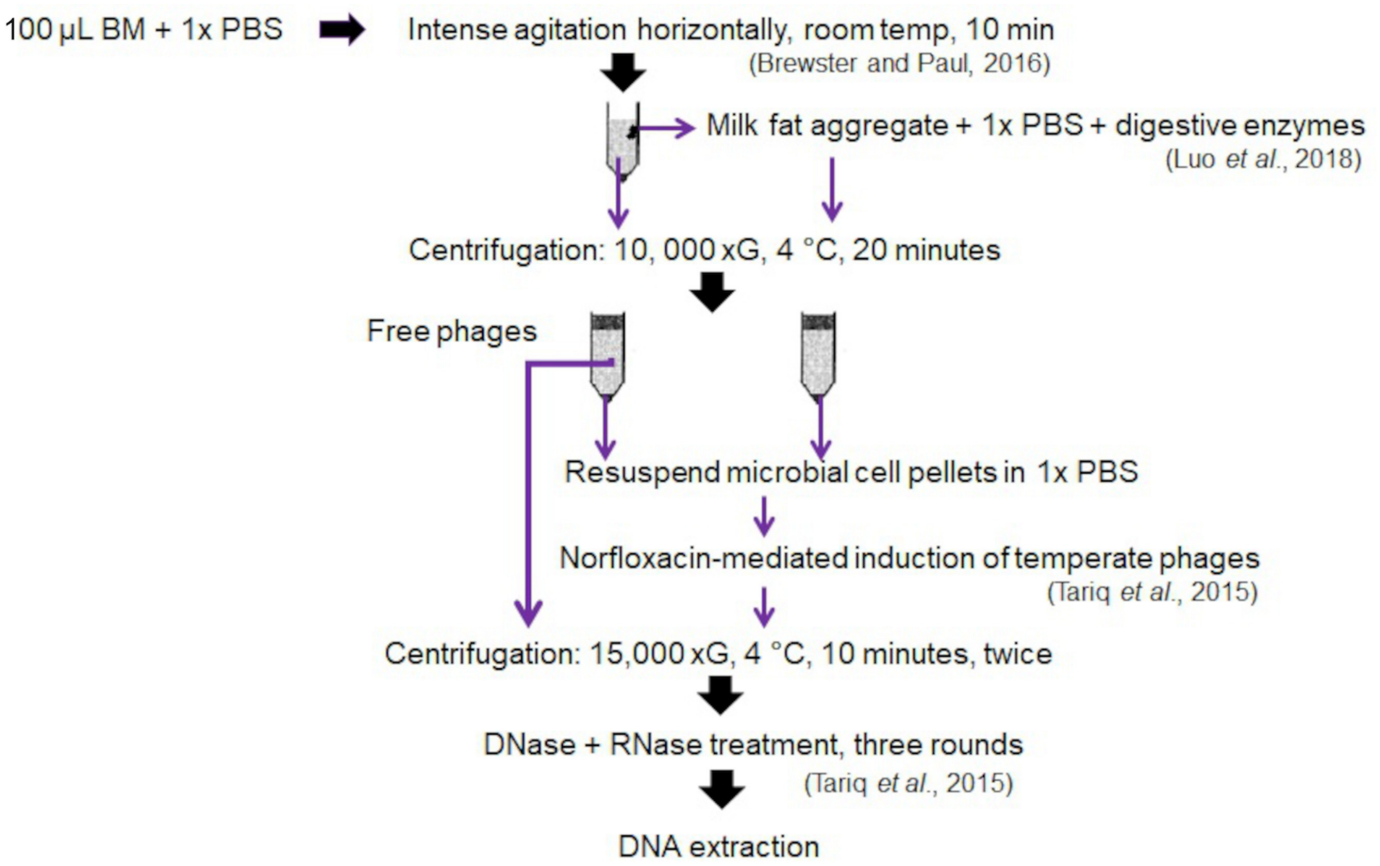
Optimized phage isolation protocol generated from the validation of different tested variables.

The absence of any impact on DNA concentration, bacteriophage richness or diversity of increasing DM sample input volume validates our optimised protocol for bacteriophage community composition from as little as 0.1 mL DM. We found hydrolysis of DM lipids with digestive enzymes, 10 K xG centrifugal separation at 4°C for 20 min and norfloxacin-mediated induction of temperate bacteriophages from the microbial cell pellets yields best results. We find no evidence for the inclusion of a freezing step prior to separation of DM lipids from the whey, nor preferential use of either QIAamp MinElute or Norgen Phage DNA isolation kits. Our optimised protocol is illustrated in Figure 4.

#### Optimized Method Application in Mother-Infant Pair Samples

To demonstrate the utility of the validated isolation methods we applied it to a small cohort of 4 mother-infant pairs. All were inpatients at the RVI, NICU, Newcastle upon Tyne Hospitals, and received expressed MM feeds, supplemented with *Labinic* probiotics (Biofloratech Ltd., UK). One BM milk and one neonatal stool sample taken within 48 h of BM feed were analysed from each mother-infant pair. Clinical details of the cohort are provided in Table 1.

**Table 1:** Recorded characteristics of clinical cohort used in pilot study.

	**Number of participants**	**Percent of total participants**
Cesarean delivery	3	75
Multiple birth	1	25
Male	2	50
Probiotic receipt	4	100
	**Median**	**Full range**
Birthweight	975	585–1400
Gestational age	28	24–30
Day of life first MM	3	2–4
Age at sampling (days)	17	7–22

*Categorical data are presented as a numerical count, plus percent of cohort. Continuous data are presented as the median and full range of data*.

BM yielded greater raw and viral read count than neonatal stools however this did not translate to a greater number or length of assembled contigs with a minimum of 5 x coverage (Supplementary Table 3). Kit and sequencing negative control samples each yielded zero contigs.

**Figure 5:**
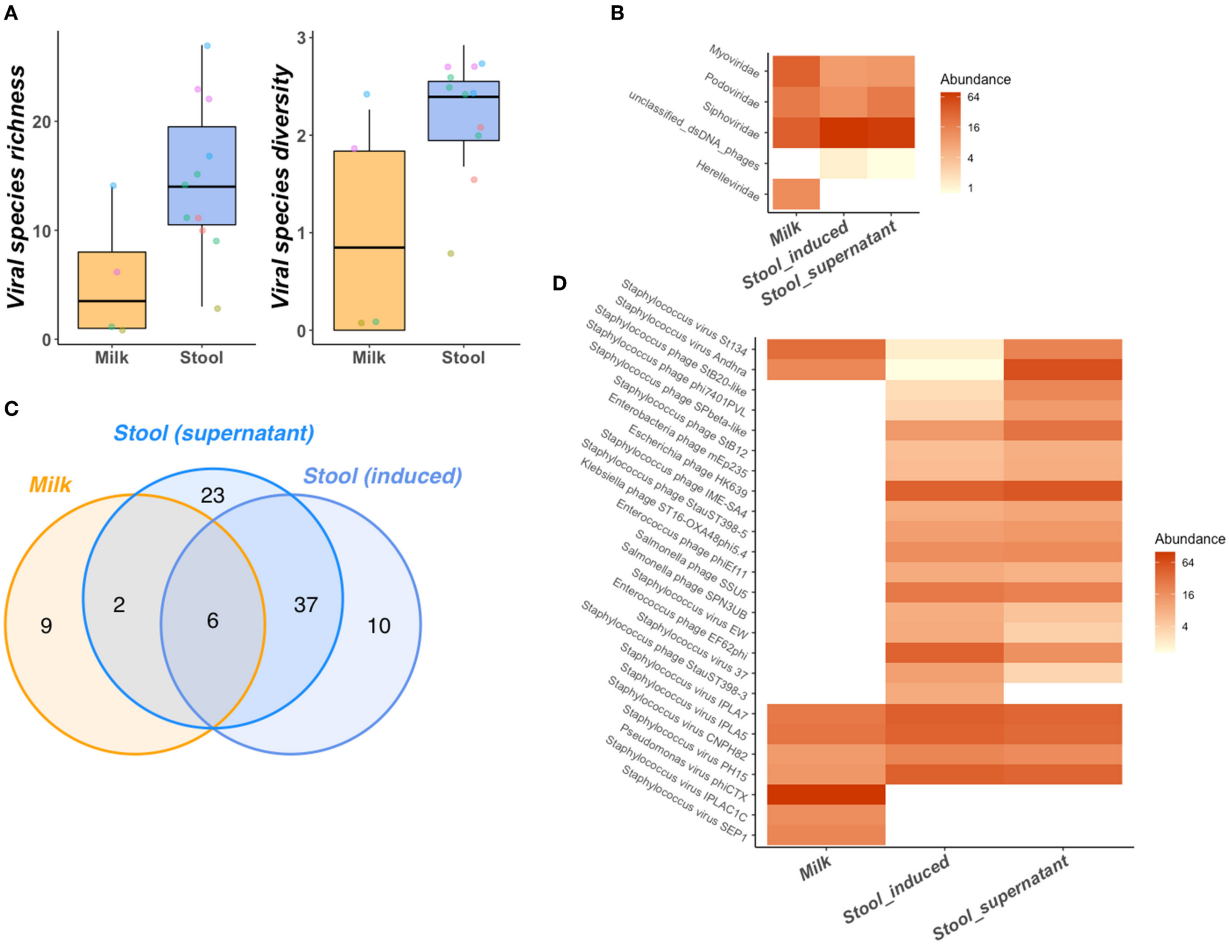
Bacteriophage community of mother-infant pair BM and stool samples. BM has lower species richness and diversity than stool **(A)** despite links between BM and neonatal stool richness and diversity as illustrated points, colored by mother-infant pair. Several bacteriophage taxonomic features are shared between BM and stool samples at the family level **(B)**. Bacteriophage species composition of BM and neonatal stool is illustrated across all species observed *via* venn diagram **(C)** and in more detail as a heatmap containing only the top 25 most abundant & prevalent bacteriophage species **(D)**. Greater relative abundance of species/families in heatmaps **(B,D)** is represented by greater color intensity [transformed on a log(2) scale].

Significantly greater bacteriophage species richness (*P* = 0.04) and diversity (*P* = 0.04) were observed in neonatal stool samples than BM (Figure 5A). A total of 78 bacteriophage species belonging to 4 families were identified in stool with only 18 bacteriophage species from 4 families identified in BM (Figure 5B). Despite these differences, greater richness and diversity in maternal BM were often observed alongside greater richness and diversity in the paired neonatal stool samples (Figure 5A). Furthermore, we identify 6 bacteriophage species that were shared between BM and neonatal stools in two different mother-infant pairs (Figures 5C,D). All 6 species were putative *Staphylococcus* infecting bacteriophage, 4 of which belonged to the *Siphoviridae* and 2 to the *Podoviridae* families. Several bacteriophage species remain exclusively identifiable in either BM or neonatal stool (Figure 5D).

## Discussion

Characterising the mechanisms of action of bioactive components in BM is key to understanding its beneficial effects. To maximise the reliability and generalisability of studies investigating these components, it is important to validate laboratory processes utilized in large-scale surveys. Despite this, few studies exist exploring methods for the isolation of complex bacteriophage communities from human BM.

This study represents a validation and optimisation of methods to reliably characterise the bacteriophage component of BM from mothers of very preterm infants by maximising the ability to observe the full diversity of bacteriophages within the sample while minimizing inherent biases, protocol cost and time. Specifically, we justify the use of very small sample volumes (as little as 0.1 mL) for very preterm BM phage community characterisation. Previous studies investigating the milk virome have utilized as much as 1.5–10 mL of BM (13–15). Reducing required sample volumes is of particular importance because preterm delivery can have a negative effect on lactogenesis and therefore salvageable volumes can be small to allow as much BM as possible to be available for the infant.

Similar to previous studies which applied viral purification protocols using CsCl density gradient ultracentrifugation on term BM (14), we identify *Mimiviridae* (a giant group of viruses) in DM from our sequencing data that were not previously reported in BM studies using micron-sized membrane filters (16). Our proposed method omits the time-consuming CsCl density gradient purification which has previously been reported to have low reproducibility and is not well-suited for high-throughput studies (45, 46).

PEG has been used as an alternative to CsCl density gradients, proving to be a cost-effective way to substantially increase nucleic acid yields and give reproducible results compared to other methods for concentrating bacteriophages from 1 to 50 mL adult human stool samples (21, 45). Importantly we did not see differences between samples when isolating phage nucleic acids from DM with PEG or our proposed method. We also did not find any increased diversity or richness of phage genera using PEG concentration protocols. This is possible because more mature individuals have greater community diversity (47, 48), requiring substantially greater sample volumes to fully characterize the overall community structure. These same volumes would be impossible to salvage from very preterm infants and mothers. This is of note as the use of PEG concentrations to purify phage particles may introduce inherent biases by preferentially selecting for specific viral particle sizes or shapes, especially in complex or mixed bacteriophage communities (49).

Our protocol enabled us to identify 6 viral families (*Adenoviridae, Baculoviridae, Herelleviridae, Papillomaviridae, Parvoviridae* and *Retroviridae*), that were not previously observed in studies using micron-sized membrane filters to explore term BM viromes. The inclusion of these families, which may well have been filtered out during unnecessary pre-processing steps highlights the impact filtration can have on observable bacteriophage communities. Indeed, filtration has been shown to reduce viral particles isolated from stools by almost 50% (50).

Previous studies of the viral composition of BM utilized isolation directly from whole or skimmed milk without upstream intervention (51–53). Association of viruses with milk lipids are often excluded in these studies. Our data reveal the presence of bacteriophages belonging to the *Inoviridae*, and eukaryotic viruses belonging to the *Iridoviridae* and *Baculoviridae* families in the lipid fraction upon hydrolysis using digestive enzymes. This indicates the presence of microbes (particularly bacteriophages) associated with milk fat globule membranes that may have been previously overlooked. Furthermore, our data suggest norfloxacin-mediated induction of lysogens present in the microbial cell pellets following centrifugal separation is essential to identify the full complement of temperate bacteriophages within communities. In addition, we demonstrate the increased speed of centrifugal separation does not improve characterisation of the temperate bacteriophage community.

Many previous studies have utilized multiple displacement amplification (MDA) to increase nucleic acid yields for sequencing. MDA has previously been reported to preferentially amplify ssDNA phages introducing bias to community compositions by artificially inflating their numbers (54–56). As such we did not include any MDA step prior to sequencing bacteriophage nucleic acids in this study but still achieve suitable nucleic acid concentrations for sequencing library preparation and identifying ssDNA phages from the *Inoviridae* family.

We incorporate nucleases prior to nucleic acid isolation and include no RT step afterward which may overlook the role of any RNA viruses in this system (46), although similar to other gut virome studies, we still identify RNA viruses, such as members of the *Retroviridae*.

This study highlights the importance of validating protocols specifically tailored to the sample type and matrices before conducting large-scale ecological surveys.

After validating a method for bacteriophage community characterisation in DM we applied this same protocol to BM and matched neonatal stool samples from mother-infant pairs highlighting six shared bacteriophage species between mother's BM and infant stools. The six shared species were all putative *Staphylococcus* infecting bacteriophages belonging to the *Siphoviridae* (4) and *Podoviridae* (2) families. Previous work by this group has identified *Staphylococcus* as the dominant member of bacterial communities of preterm infant's stool and mother's BM during early life (8, 57). Other highly abundant bacteriophages identified in this study, include *Escherichia, Enterobacteria* and *Klebsiella* phages. Although the host specificity of these phages is purely based on sequencing data, rather than host-range studies these results align with those of Liang et al. (16) who identified bacteriophage colonizing infant guts in the immediate post-natal period to be mostly comprised of temperate phages induced from bacterial community members. Previous studies have yielded conflicting results regarding the vertical transmission of bacteriophages between mothers and infants (12–15). This study is the first to our knowledge to provide evidence for the potential of this phenomenon in very preterm infants who, importantly, receive much smaller feed volumes than term infants.

Despite being adopted in previous studies as a measure of viral recovery efficiency (58), our method validation data suggest DNA yield is a poor predictor of this. In two experiments the protocol yielding the greatest DNA concentration did not translate to greater bacteriophage richness or diversity. This is likely because despite multiple bacteriophage isolation and purification steps we still cannot confirm extracted nucleic acids are free from sources of other contaminating organisms. Alternative methods could spike known compositions of viral particles into sterile matrices, however, acquisition of sterile matrices with similar complexity to human milk proves extremely difficult. Our selection of pasteurized DM was initially intended to facilitate this, but our data demonstrate the persistence of bacteriophage nucleic acids despite pasteurisation. In addition, bacteriophage communities in DM and BM are not well studied so it is difficult to select bacteriophage species/strains for effective validation purposes (45).

The persistence of bacteriophage nucleic acids post-pasteurisation of DM highlights the need for further work to discriminate between viable and non-viable bacteriophage communities associated with the very preterm infant GIT.

This study developed an optimised protocol for the isolation of a broad range of bacteriophages from small volumes of BM samples. Using the validated bacteriophage isolation protocol, we have successfully identified viral communities in pasteurized DM, as well as in bio-banked preterm BM. Shared bacteriophage species between BM and stool samples from the same mother-infant pairs were also identified. These data will serve as baseline information toward future studies to develop greater understanding of how bacteriophages may be implicated in the development of the preterm gut microbiome.

## Data Availability Statement

The original contributions presented in the study are publicly available. This data can be found here: https://www.ebi.ac.uk/ena, PRJEB49989.

## Ethics Statement

The studies involving human participants were reviewed and approved by NRES Committee North East–Newcastle and North Tyneside 2 (10/H0908/39). Written informed consent to participate in this study was provided by the participants' legal guardian/next of kin.

## Author Contributions

WY, AN, SB, and DS conceived the method validation study. WY performed laboratory and bioinformatic work for the validation study. GY and DS conceived the application in the mother-infant pairs study. GY performed laboratory and bioinformatic work for this. AN sequenced the libraries. JB and NE enrolled participants in the clinical study and arranged and carried out sampling. GY and WY wrote the manuscript. All authors reviewed and approved the manuscript before submission.

## Funding

This work was funded by Children's Charity Action Medical Research on grant number GN2730.

## Conflict of Interest

NE and JB declare research funding paid to their employing institution from Prolacta Biosciences US, and Danone Early Life Nutrition, and both declare lecture honoraria from Nestle Nutrition Institute. The remaining authors declare that the research was conducted in the absence of any commercial or financial relationships that could be construed as a potential conflict of interest.

## Publisher's Note

All claims expressed in this article are solely those of the authors and do not necessarily represent those of their affiliated organizations, or those of the publisher, the editors and the reviewers. Any product that may be evaluated in this article, or claim that may be made by its manufacturer, is not guaranteed or endorsed by the publisher.
