# Supplementary material for: Optimisation and Application of a Novel Method to Identify Bacteriophages in Maternal Milk and Infant Stool Identifies Host-Phage Communities Within Preterm Infant Gut
Source: Front Pediatr. 2022 Apr 26;10:856520. doi: 10.3389/fped.2022.856520 (PMC9087270; doi:10.3389/fped.2022.856520)
Supplement: Supplementary file 1 [file Data_Sheet_1.DOCX]

Supplementary materials: Optimisation and application of a novel method to identify bacteriophage in maternal milk and infant stool identifies host-phage communities within preterm infant gut

Supplementary figure 1: Flowchart outlining experimental protocol for centrifugal sedimentation of microbial cells

Supplementary figure 2: Flowchart outlining experimental protocol for bacteriophage isolation including hydrolysis

Supplementary figure 3: Flowchart outlining experimental protocol for separated milk lipid processing

Supplementary figure 4: Flowchart outlining experimental protocol for bacteriophage isolation excluding hydrolysis

Supplementary table 1: Experimental conditions applied to each sample included in protocol validation study

Supplementary table 2: Composition of bacteriophage communities in DM samples used for isolation protocol validation. Table shows families and genera present in at least 50% of samples.

Supplementary table 3: Read distribution in clinical pilot study samples

Supplementary table 4: Impact of commercially available nucleic acid extraction kits on bacteriophage community composition

Supplementary figure 5: Impact of commercially available nucleic acid extraction kits on bacteriophage community composition

Supplementary figure 1: Flowchart outlining experimental protocol for centrifugal sedimentation of microbial cells


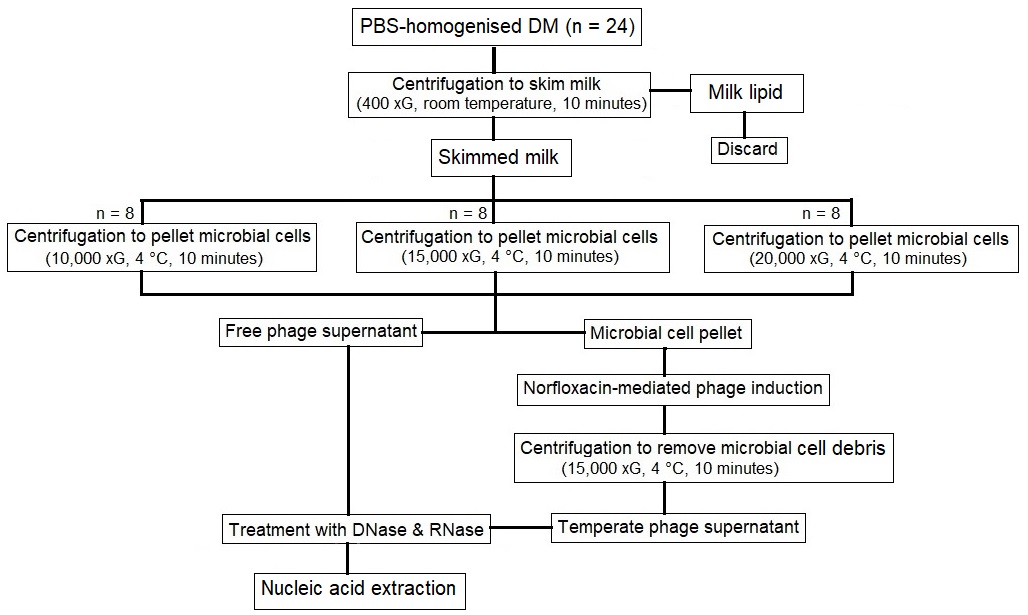


Supplementary figure 2: Flowchart outlining experimental protocol for bacteriophage isolation including hydrolysis


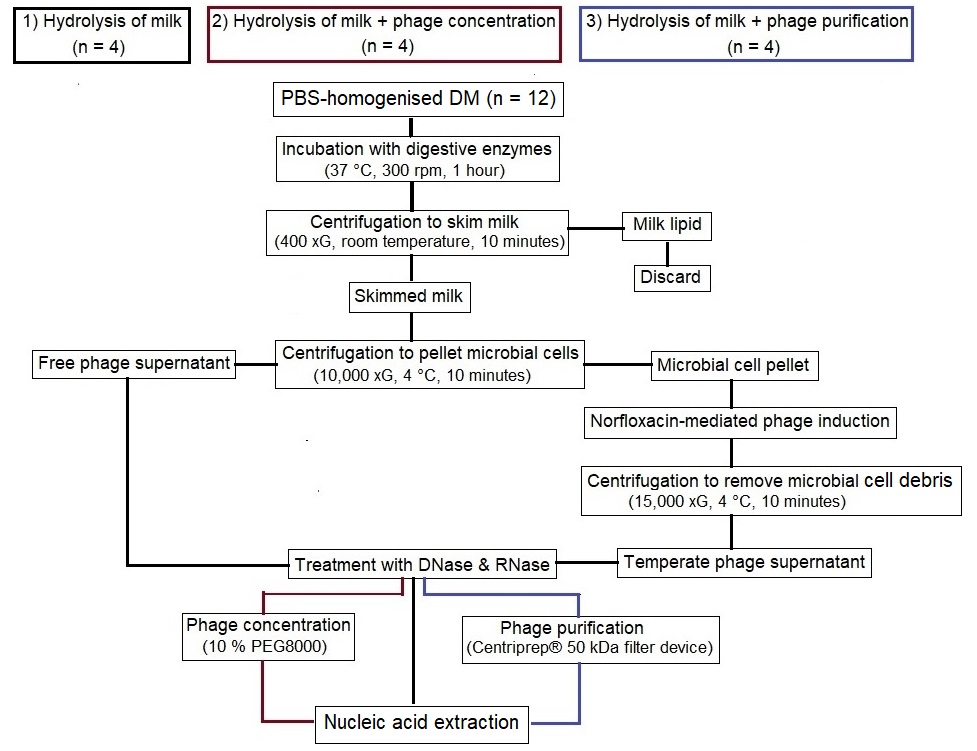


Supplementary figure 3: Flowchart outlining experimental protocol for separated milk lipid processing


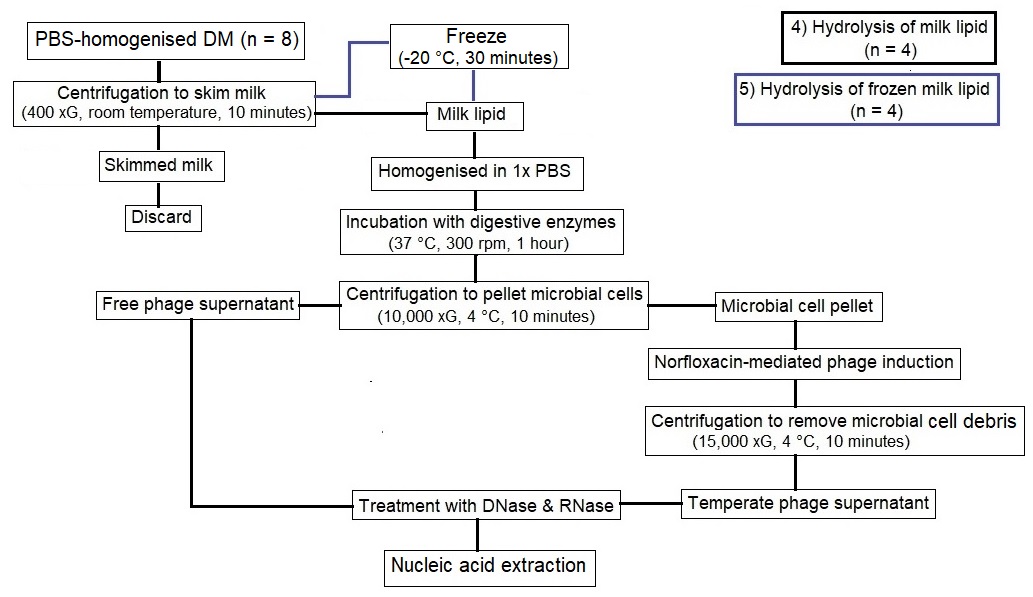


Supplementary figure 4: Flowchart outlining experimental protocol for bacteriophage isolation excluding hydrolysis


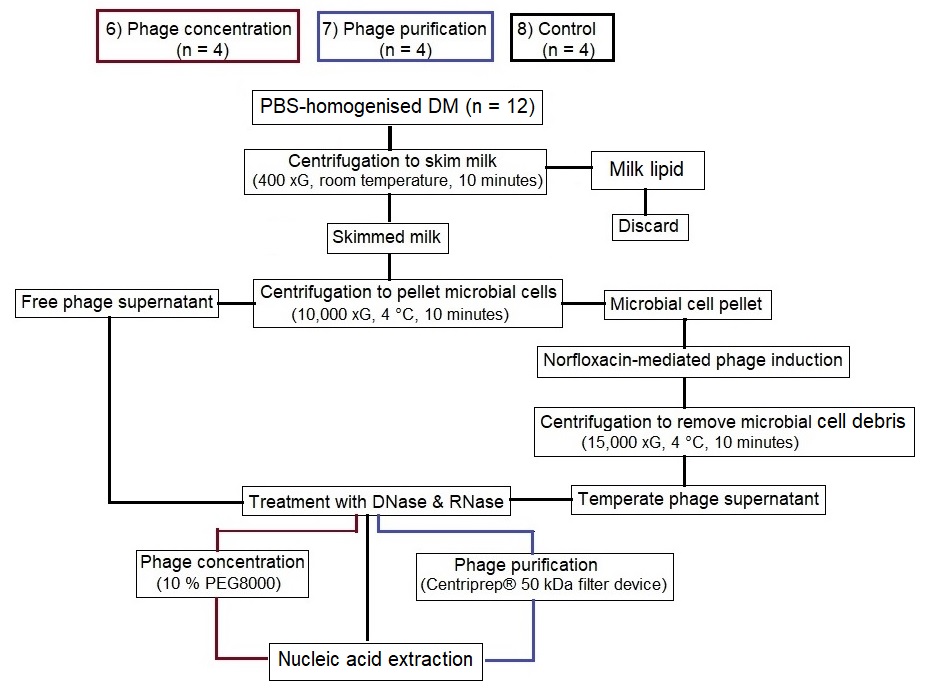


Supplementary table 1: Experimental conditions applied to each sample included in protocol validation study

| **Sample** | **Sample volume  (mL)** | **Milk fraction** | **Phage type** | **Centrifuge speed (xG)** | **Optimisation** |
| --- | --- | --- | --- | --- | --- |
| S1 | 0.2 | Milk | Induced | 10k | Gastric |
| S2 | 0.4 | Milk | Induced | 10k | Gastric |
| S3 | 0.2 | Milk | Free | 10k | Gastric |
| S4 | 0.4 | Milk | Free | 10k | Gastric |
| S5 | 0.2 | Milk_fat | Induced | 10k | Gastric |
| S6 | 0.4 | Milk_fat | Induced | 10k | Gastric |
| S7 | 0.2 | Milk_fat | Free | 10k | Gastric |
| S8 | 0.4 | Milk_fat | Free | 10k | Gastric |
| S9 | 0.6 | Milk | Induced | 10k | Gastric |
| S10 | 0.6 | Milk | Free | 10k | Gastric |
| S11 | 0.2 | Milk | Induced | 10k | Gastric+PEG8000 |
| S12 | 0.4 | Milk | Induced | 10k | Gastric+PEG8000 |
| S13 | 0.2 | Milk | Free | 10k | Gastric+PEG8000 |
| S14 | 0.4 | Milk | Free | 10k | Gastric+PEG8000 |
| S15 | 0.2 | Milk | Induced | 10k | PEG8000 |
| S16 | 0.4 | Milk | Induced | 10k | PEG8000 |
| S17 | 0.2 | Milk | Free | 10k | PEG8000 |
| S18 | 0.4 | Milk | Free | 10k | PEG8000 |
| S19 | 0.6 | Milk | Induced | 10k | Gastric+PEG8000 |
| S20 | 0.6 | Milk | Free | 10k | Gastric+PEG8000 |
| S21 | 0.2 | Milk | Induced | 10k | Gastric+Centriprep |
| S22 | 0.4 | Milk | Induced | 10k | Gastric+Centriprep |
| S23 | 0.2 | Milk | Free | 10k | Gastric+Centriprep |
| S24 | 0.4 | Milk | Free | 10k | Gastric+Centriprep |
| S25 | 0.2 | Milk | Induced | 10k | Centriprep |
| S26 | 0.4 | Milk | Induced | 10k | Centriprep |
| S27 | 0.2 | Milk | Free | 10k | Centriprep |
| S28 | 0.4 | Milk | Free | 10k | Centriprep |
| S29 | 0.6 | Milk | Induced | 10k | Gastric+PEG8000+Centriprep |
| S30 | 0.6 | Milk | Free | 10k | Gastric+PEG8000+Centriprep |
| S31 | 0.1 | Milk | Induced | 10k | None |
| S32 | 0.1 | Milk | Free | 10k | None |
| S33 | 0.1 | Milk | Induced | 15k | None |
| S34 | 0.2 | Milk | Induced | 15k | None |
| S35 | 0.4 | Milk | Induced | 15k | None |
| S36 | 0.6 | Milk | Induced | 15k | None |
| S37 | 0.1 | Milk | Free | 15k | None |
| S38 | 0.2 | Milk | Free | 15k | None |
| S39 | 0.4 | Milk | Free | 15k | None |
| S40 | 0.6 | Milk | Free | 15k | None |
| S41 | 0.1 | Milk | Induced | 20k | None |
| S42 | 0.2 | Milk | Induced | 20k | None |
| S43 | 0.4 | Milk | Induced | 20k | None |
| S44 | 0.6 | Milk | Induced | 20k | None |
| S45 | 0.1 | Milk | Free | 20k | None |
| S46 | 0.2 | Milk | Free | 20k | None |
| S47 | 0.4 | Milk | Free | 20k | None |
| S48 | 0.6 | Milk | Free | 20k | None |
| S49 | 0.1 | Frozen_milk | Induced | 10k | None |
| S50 | 0.2 | Frozen_milk | Induced | 10k | None |
| S51 | 0.4 | Frozen_milk | Induced | 10k | None |
| S52 | 0.1 | Frozen_milk | Free | 10k | None |
| S53 | 0.2 | Frozen_milk | Free | 10k | None |
| S54 | 0.4 | Frozen_milk | Free | 10k | None |
| S55 | 0.1 | Frozen_milk_fat | Induced | 10k | Gastric |
| S56 | 0.2 | Frozen_milk_fat | Induced | 10k | Gastric |
| S57 | 0.4 | Frozen_milk_fat | Induced | 10k | Gastric |
| S58 | 0.1 | Frozen_milk_fat | Free | 10k | Gastric |
| S59 | 0.2 | Frozen_milk_fat | Free | 10k | Gastric |
| S60 | 0.4 | Frozen_milk_fat | Free | 10k | Gastric |
| S61 | 0.2 | Milk | Induced | 10k | None |
| S62 | 0.4 | Milk | Induced | 10k | None |
| S63 | 0.6 | Milk | Induced | 10k | None |
| S64 | 0.2 | Milk | Free | 10k | None |
| S65 | 0.4 | Milk | Free | 10k | None |
| S66 | 0.6 | Milk | Free | 10k | None |
| S67 | 0.2 | Milk | Induced | 10k | None |
| S68 | 0.4 | Milk | Induced | 10k | None |
| S69 | 0.6 | Milk | Induced | 10k | None |
| S70 | 0.2 | Milk | Free | 10k | None |
| S71 | 0.4 | Milk | Free | 10k | None |
| S72 | 0.6 | Milk | Free | 10k | None |
| S73 | 0.2 | Milk | Induced | 10k | None |
| S74 | 0.4 | Milk | Induced | 10k | None |
| S75 | 0.6 | Milk | Induced | 10k | None |
| S76 | 0.2 | Milk | Free | 10k | None |
| S77 | 0.4 | Milk | Free | 10k | None |
| S78 | 0.6 | Milk | Free | 10k | None |

Supplementary table 2: Composition of bacteriophage communities in DM samples used for isolation protocol validation. Table shows families and genera present in at least 50% of samples.

| **FAMILY** | **GENUS (% presence)** |
| --- | --- |
| **Herelleviridae** | *Sepunavirus (79)* |
|  | *Cytomegalovirus (84)* |
|  |  |
| **Myoviridae** | *Hpunavirus (51)* |
|  | *Tequatrovirus (71)* |
|  | *unclassified Myoviridae (93)* |
|  | *Peduovirus (94)* |
|  | *Punavirus (95)* |
|  |  |
| **Podoviridae** | *Uetakevirus (54)* |
|  | *unclassified Podoviridae (54)* |
|  | *Lederbergvirus (69)* |
|  | *Rosenblumvirus (85)* |
|  | *unclassified Picovirinae (91)* |
|  |  |
| **Poxviridae** | *unclassified Chordopoxvirinae (95)* |
|  |  |
| **Retroviridae** | *Human endogenous retroviruses (76)* |
|  |  |
| **Siphoviridae** | *Efquatrovirus (51)* |
|  | *Roufvirus (59)* |
|  | *Sextaecvirus (66)* |
|  | *Spbetavirus (66)* |
|  | *Pahexavirus (74)* |
|  | *Biseptimavirus (78)* |
|  | *Vieuvirus (80)* |
|  | *Nickievirus (85)* |
|  | *Nonanavirus (93)* |
|  | *Chivirus (95)* |
|  | *Lambdavirus (95)* |
|  | *Phifelvirus (95)* |
|  | *unclassified Siphoviridae (99)* |
|  |  |
| **unclassified Caudovirales** | *unclassified Caudovirales (68)* |
|  |  |
| **unclassified phages** | *unclassified dsDNA phages (59)* |

Supplementary table 3: Read distribution in clinical pilot study samples

|  | ***Raw reads*** | ***Viral reads*** | ***Contigs*** | ***Contig length*** |
| --- | --- | --- | --- | --- |
| ***Milk*** |  |  |  |  |
| *Median (N50)* | 5925281.5 | 4474882.5 | 29.5 | 782.6904762 |
| *3rd Quartile (N75)* | 6469394.75 | 4734614.75 | 50 | 1553.312741 |
| ***Stool*** |  |  |  |  |
| *Median (N50)* | 3010485 | 2619886 | 150 | 2944.695652 |
| *3rd Quartile (N75)* | 3246847 | 2898654 | 213.5 | 5171.142857 |

Supplementary table 4: Impact of commercially available nucleic acid extraction kits on bacteriophage community composition

|  |  |  | ***Roche High Pure Viral Nucleic Acid Kit*** | ***QIAamp MinElute Virus Spin Kit*** | ***Norgen Phage DNA Isolation Kit*** |
| --- | --- | --- | --- | --- | --- |
| ***Isolated DNA conc (ng/µL)*** | |  |  |  |  |
|  |  | *Median* | 1.75 | 2.22 | 0.65 |
|  |  | *IQR* | 1.71 - 1.76 | 1.99 - 2.52 | 0.38 - 1.00 |
|  |  | *KW* | *(KW: χ2 = 12.24, df = 1, P = 0.002)* | | |
| ***Bacteriophage richness*** | |  |  |  |  |
|  |  | *Median* | 22 | 26 | 26 |
|  |  | *IQR* | 20 - 24 | 25 - 27 | 26 - 27 |
|  |  | *KW* | (KW: χ2 = 5.51, df = 1, P = 0.06) | | |
| ***Shannon diversity*** | |  |  |  |  |
|  |  | *Median* | 2.74 | 3.11 | 3.15 |
|  |  | *IQR* | 2.59 - 2.91 | 3.05 - 3.17 | 3.12 - 3.20 |
|  |  | *KW* | *(KW: χ2 = 10.15, df = 2, P = 0.006)* | | |
| ***Raw reads*** |  |  |  |  |  |
|  |  | *Median* | 94866 | 534829 | 529187 |
|  |  | *IQR* | 70724 - 285232 | 426947 - 554761 | 431838 - 705667 |
|  |  | *KW* | *(KW: χ2 = 8.67, df = 2, P = 0.01)* | | |
| ***High quality reads*** | |  |  |  |  |
|  |  | *Median* | 86104 | 440789 | 475976 |
|  |  | *IQR* | 60041 - 258695 | 361210 - 484213 | 372902 - 639439 |
|  |  | *KW* | *(KW: χ2 = 9.01, df = 2, P = 0.01)* | | |
| ***Proportion of reads mapped to bacteria*** | | |  |  |  |
|  |  | *Median* | 0.119 | 0.127 | 0.078 |
|  |  | *IQR* | 0.071 - 0.184 | 0.081 - 0.169 | 0.071 - 0.114 |
|  |  | *KW* | (KW: χ2 = 1.63, df = 2, P = 0.44) | | |
| ***ViromeQC enrichment score*** | | |  |  |  |
|  |  | *Median* | 6.89 | 5.90 | 9.00 |
|  |  | *IQR* | 3.82 - 9.88 | 4.14 - 8.69 | 6.40 - 9.84 |
|  |  | *KW* | (KW: χ2 = 1.72, df = 2, P = 0.42) | | |

Supplementary figure 5: Impact of commercially available nucleic acid extraction kits on bacteriophage community composition


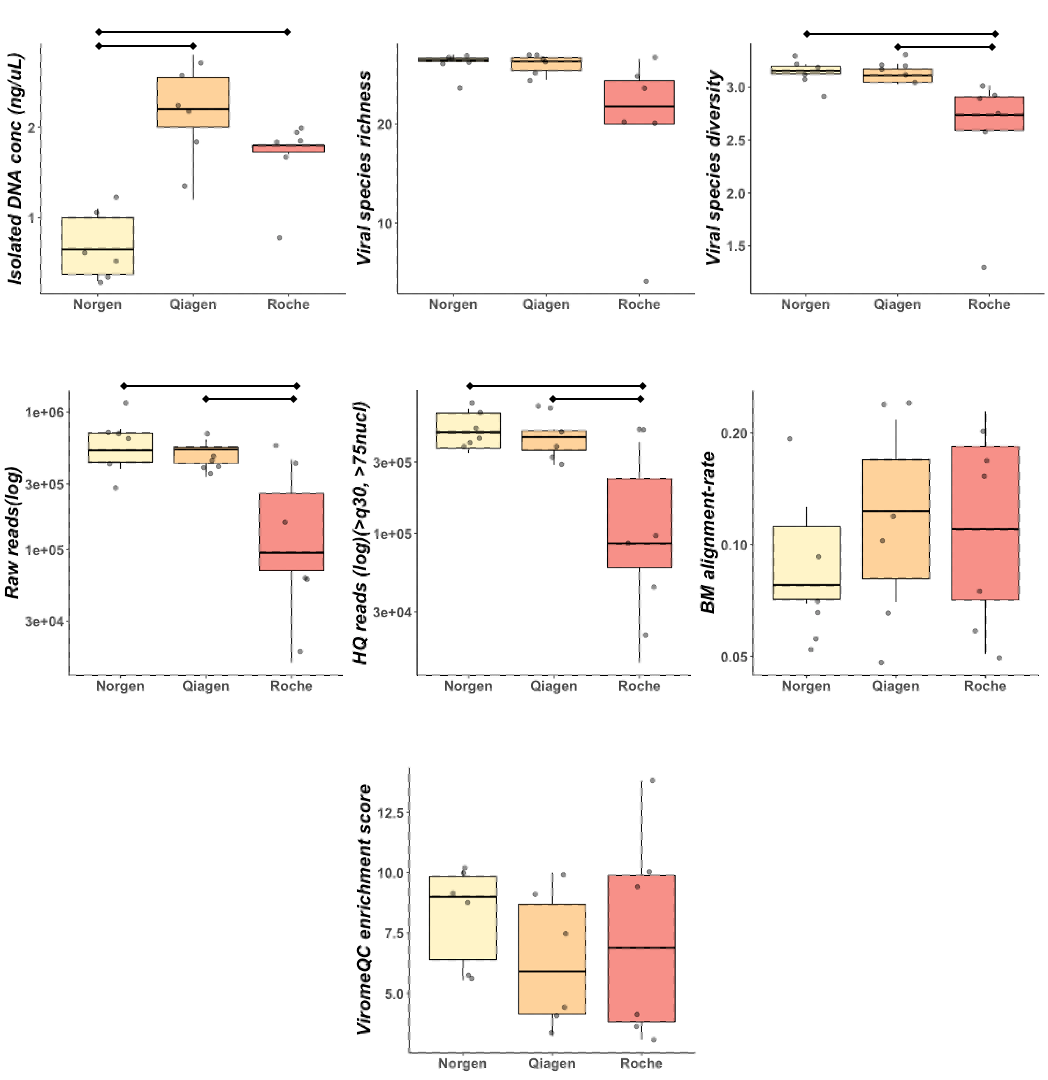


Figure 4: Comparisons of DNA concentration, genus richness, Shannon diversity, raw reads, high quality reads, bacterial read content, and virome enrichment scores between different viral DNA extraction kits. Boxes represent the 1st and 3rd quartiles. Middle lines represent the median. Whiskers extend to the full range of data. Points represent individual samples. Significant differences between parameters identified by pairwise Mann-Whitney test are highlighted with lines above groups.
